# Immune regulator IRF1 contributes to ZBP1-, AIM2-, RIPK1-, and NLRP12-PANoptosome activation and inflammatory cell death (PANoptosis)

**DOI:** 10.1016/j.jbc.2023.105141

**Published:** 2023-08-07

**Authors:** Bhesh Raj Sharma, Rajendra Karki, Yetirajam Rajesh, Thirumala-Devi Kanneganti

**Affiliations:** Department of Immunology, St Jude Children's Research Hospital, Memphis, Tennessee, USA

**Keywords:** inflammasome, pyroptosis, apoptosis, necroptosis, PANoptosis, PANoptosome, inflammatory cell death, NLRP3, caspase-8, influenza virus, herpes simplex virus, gasdermin, caspase, interferon, innate immunity

## Abstract

The innate immune system provides the first line of defense against pathogens and cellular insults and is activated by pattern recognition receptors sensing pathogen- or damage-associated molecular patterns. This activation can result in inflammation *via* cytokine release as well as the induction of lytic regulated cell death (RCD). Innate immune signaling can also induce the expression of interferon regulatory factor 1 (IRF1), an important molecule in regulating downstream inflammation and cell death. While IRF1 has been shown to modulate some RCD pathways, a comprehensive evaluation of its role in inflammatory cell death pathways is lacking. Here, we examined the role of IRF1 in cell death during inflammasome and PANoptosome activation using live cell imaging, Western blotting, and ELISA in primary murine macrophages. IRF1 contributed to the induction of ZBP1- (Z-DNA binding protein 1), AIM2- (absent in melanoma-2), RIPK1- (receptor interacting protein kinase 1), and NLRP12 (NOD-like receptor family, pyrin domain–containing 12)-PANoptosome activation and PANoptosis. Furthermore, IRF1 regulated the cell death under conditions where inflammasomes, along with caspase-8 and RIPK3, act as integral components of PANoptosomes to drive PANoptosis. However, it was dispensable for other inflammasomes that form independent of the PANoptosome to drive pyroptosis. Overall, these findings define IRF1 as an upstream regulator of PANoptosis and suggest that modulating the activation of molecules in the IRF1 pathway could be used as a strategy to treat inflammatory and infectious diseases associated with aberrant inflammatory cell death.

The innate immune system detects pathogens and cellular stressors to act as the body’s first line of defense against infection and disease. Pathogen-associated molecular patterns (PAMPs) and damage-associated molecular patterns (DAMPs) activate innate immune sensors called pattern recognition receptors to induce a variety of downstream signaling events, including the production of inflammatory cytokines and interferons (IFNs), as well as the activation of regulated cell death (RCD). RCD is an essential component of the innate immune response to destroy the replicative niche of pathogens and remove unwanted cells to maintain homeostasis (1). RCD pathways can be divided into two categories, nonlytic and lytic. Apoptosis is the canonical nonlytic pathway, whereas the inflammatory pyroptosis and necroptosis are the best characterized lytic pathways. These pathways were historically considered independent, but extensive crosstalk among their molecular components has now been identified (2, 3, 4, 5, 6, 7, 8, 9, 10, 11, 12, 13), leading to the conceptualization of PANoptosis. PANoptosis is a unique innate immune lytic, inflammatory RCD pathway that is driven by caspases and receptor interacting protein kinases (RIPKs) and regulated by multiprotein PANoptosome complexes that integrate components from other cell death pathways (14). To date, four PANoptosome complexes have been molecularly characterized, the Z-DNA binding protein 1 (ZBP1)-PANoptosome (5, 11, 15), AIM2 (absent in melanoma-2)-PANoptosome (16), RIPK1-PANoptosome (17), and NLRP12 (NLR family, pyrin domain-containing 12)-PANoptosome (18).

Studies on the upstream signaling regulating PANoptosis have identified a role for IFN signaling in many cases, particularly in the context of the ZBP1-PANoptosome (5, 19). A key component of IFN signaling is the activation of IFN regulatory factors (IRFs), which are transcription factors that modulate downstream signaling events. One such IRF is IRF1 (20, 21), which plays a role in cell death *via* its transcriptional regulation of innate immune gene expression (22). Furthermore, IRF1 was first identified to be an inflammasome regulator in the context of the AIM2 inflammasome (23). IRF1 induction can be detrimental and contribute to chronic inflammatory diseases (24, 25), but it can also be beneficial and aid in pathogen elimination during *Francisella*, influenza A virus (IAV), and other RNA virus infections (5, 23, 26), and provides protection from colorectal cancer (27). The IRF1-mediated protection from colorectal cancer occurs through increased PANoptosis, suggesting there may be a mechanistic link between IRF1 and the regulation of PANoptosis. Similarly, PANoptosis can be driven by the combination of tumor necrosis factor (TNF) and IFNγ, and human colon cancer cells deficient in IRF1 are resistant to TNF plus IFNγ-induced PANoptosis (28). Furthermore, TNF and IFNγ released during severe acute respiratory syndrome coronavirus 2 (SARS-CoV-2) infection induce PANoptosis through the Janus kinase–IRF1 signaling axis to drive cytokine storm and inflammatory pathology (29). In addition, a recent study showed that IRF1 is an upstream regulator of NLRP12-PANoptosome activation in response to heme plus PAMPs or TNF, such as in the context of hemolytic disease (18). IFN signaling has also been shown to regulate key proteins required for PANoptosis. IRF1 upregulates ZBP1 to form the ZBP1-PANoptosome and drive PANoptotic cell death during IAV infection (5). In addition, in a context-dependent manner, IFN signaling regulates inflammasomes such as the AIM2 and NLRP3 inflammasomes (5, 19, 23), and these inflammasomes can act as integral components of PANoptosomes to drive PANoptosis (11, 15, 16).

Overall, IFN signaling has been associated with inflammasomes that act as integral components of PANoptosomes, IRF1 is an upstream regulator of the NLRP12-PANoptosome (18), and IRF1—but not other IRFs—serves as a key regulator of PANoptosis in response to TNF plus IFNγ (29); however, whether IRF1 regulates PANoptosis more generally in response to other triggers and in different PANoptosome complexes (ZBP1-, AIM2-, and RIPK1-PANoptosomes) remains unknown. Here, we provide a comprehensive assessment of the role of IRF1 in inflammasome activation and inflammatory cell death. IRF1 regulated PANoptosis in response to infection with *Francisella novicida*, herpes simplex virus type 1 (HSV1), and IAV and in response to PANoptosis-inducing ligands such as IFN plus KPT-330 or heme plus PAMPs or TNF, and homeostatic disruptions through the inhibition of TAK1 (transforming growth factor-β activating protein kinase 1) with lipopolysaccharide (LPS) priming. However, we show that IRF1 was dispensable in the regulation of cell death in response to inflammasome triggers that are not associated with PANoptosis, such as *Escherichia coli* infection. Overall, our study clarifies the key role of IRF1 in inflammatory cell death, PANoptosis, and suggests that IRF1 regulation is a differentiating factor between inflammasome formation for pyroptosis *versus* inflammasome formation to act as an integral component of a PANoptosome, along with caspase-8 and RIPK3, to drive PANoptosis. Therefore, targeting the molecules in the IRF1 pathway could serve as a therapeutic strategy to improve patient outcomes in diseases where PANoptosis drives inflammatory pathology.

## Results

### IRF1 is dispensable for cell death in response to infection with classical inflammasome-inducing pathogens

**Figure 1:**
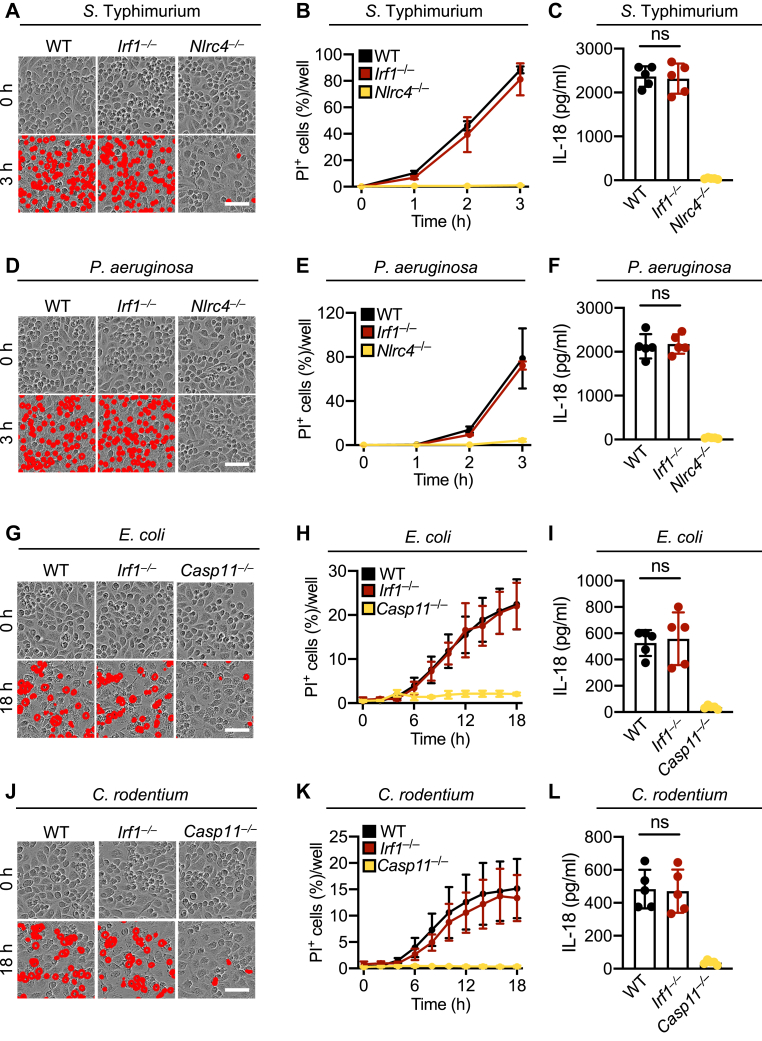
**IRF1 is dispensable for cell death in response to infection with classical inflammasome-inducing pathogens.***A*–*F*, representative images of cell death and IL-18 release in WT, *Irf1*^*−/−*^, and *Nlrc4*^*−/−*^ bone marrow–derived macrophages (BMDMs) at 3 h postinfection with *Salmonella* Typhimurium (1 MOI) (*A* and *C*) or *Pseudomonas aeruginosa* (2 MOI) (*D* and *F*). Real-time analysis of cell death in WT, *Irf1*^*−/−*^, *and Nlrc4*^*−/−*^ BMDMs following infection with *S.* Typhimurium (1 MOI) (*B*) or *P. aeruginosa* (2 MOI) (*E*). *G*–*L*, representative images of cell death and IL-18 release in WT, *Irf1*^*−/−*^, and *Casp11*^*−/−*^ BMDMs at 18 h postinfection with *Escherichia coli* (20 MOI) (*G* and *I*) or *Citrobacter rodentium* (20 MOI) (*J* and *L*). Real-time analysis of cell death in WT, *Irf1*^*−/−*^, and *Casp11*^*−/−*^ BMDMs following infection with *E. coli* (20 MOI) (*H*) or *C. rodentium* (20 MOI) (*K*). Data are representative of at least three independent biological replicates (*A* and *B*; *D* and *E*; *G* and *H*; *J* and *K*), and individual datapoints from independent biological replicates are shown (*C*, *F*, *I*, and *L*). Scale bar is representative of 50 μm. IL-18, interleukin 18; IRF1, interferon regulatory factor 1; MOI, multiplicity of infection; ns, not significant.

IRF1 plays a critical role in innate immune activation and host defense against common pathogens, including many bacteria (22). To comprehensively understand the role of IRF1 in cell death in response to bacteria that are known to activate the inflammasome, we infected WT and IRF1-deficient bone marrow–derived macrophages (BMDMs) with *Salmonella* Typhimurium and *Pseudomonas aeruginosa*, which activate the NLRC4 inflammasome (30, 31). Similar levels of cell death and interleukin 18 (IL-18) release were observed in WT and IRF1-deficient BMDMs in response to infection with *S.* Typhimurium (Fig. 1, *A*–*C*) and *P. aeruginosa* (Fig. 1, *D*–*F*), suggesting that IRF1 is dispensable for cell death and IL-18 release under these conditions. In contrast, BMDMs deficient in NLRC4 had no cell death and significantly reduced IL-18 release in response to these infections (Fig. 1, *A*–*F*).

Next, we evaluated the role of IRF1 in mediating cell death in response to infection with *E. coli* and *Citrobacter rodentium*, which activate the noncanonical NLRP3 inflammasome through caspase-11 activation. Again, similar levels of cell death and IL-18 release were observed in WT and IRF1-deficient BMDMs in response to infection with *E. coli* (Fig. 1, *G*–*I*) and *C. rodentium* (Fig. 1, *J*–*L*), whereas BMDMs deficient in caspase-11 had no cell death and impaired IL-18 release in response to these infections (Fig. 1, *G*–*L*). Together, these data suggest that IRF1 is dispensable for inflammasome activation and cell death in response to infection with both NLRC4 inflammasome–activating pathogens as well as caspase-11–activating pathogens.

### IRF1 is dispensable for cell death in response to classical inflammasome-inducing ligands

**Figure 2:**
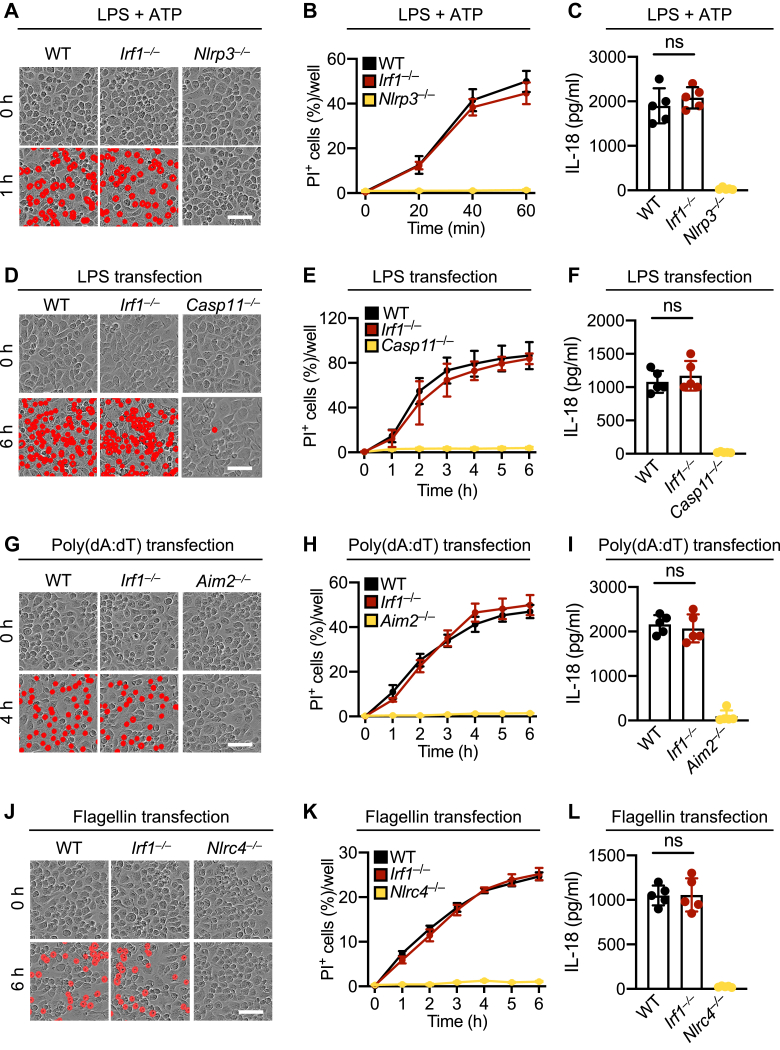
**IRF1 is dispensable for cell death in response to classical inflammasome-inducing ligands.***A*–*C*, representative images of cell death and IL-18 release in WT, *Irf1*^*−/−*^, and *Nlrp3*^*−/−*^ bone marrow–derived macrophages (BMDMs) at 1 h after stimulation with LPS plus ATP (*A* and *C*) and real-time analysis of cell death (*B*). *D*–*F*, representative images of cell death and IL-18 release in WT, *Irf1*^*−/−*^, and *Casp11*^*−/−*^ BMDMs at 6 h post-transfection with LPS (*D* and *F*) and real-time analysis of cell death (*E*). *G*–*I*, representative images of cell death and IL-18 release in WT, *Irf1*^*−/−*^, and *Aim2*^*−/−*^ BMDMs at 4 h post-transfection with poly(dA:dT) (*G* and *I*) and real-time analysis of cell death (*H*). *J*–*L*, representative images of cell death and IL-18 release in WT, *Irf1*^*−/−*^, and *Nlrc4*^*−/−*^ BMDMs at 6 h post-transfection with flagellin (*J* and *L*) and real-time analysis of cell death (*K*). Data are representative of at least three independent biological repeats (*A* and *B*; *D* and *E*; *G* and *H*; *J* and *K*), and individual datapoints from independent biological replicates are shown (*C*, *F*, *I*, and *L*). Scale bar is representative of 50 μm. IL-18, interleukin 18; IRF1, IFN regulatory factor 1; LPS, lipopolysaccharide; ns, not significant.

To assess the role of IRF1 in ligand-induced and inflammasome-mediated cell death, we analyzed cell death in WT and IRF1-deficient BMDMs in response to stimulation with LPS plus ATP (canonical NLRP3 inflammasome–activating ligands), LPS transfection (caspase-11–activating ligand), poly(dA:dT) transfection (AIM2-activating ligand), and flagellin transfection (NLRC4-activating ligand). We found similar levels of cell death and IL-18 release in WT and IRF1-deficient BMDMs in response to LPS plus ATP treatment (Fig. 2, *A*–*C*), as well as transfection with LPS (Fig. 2, *D*–*F*), poly(dA:dT) (Fig. 2, *G*–*I*), and flagellin (Fig. 2, *J*–*L*), whereas loss of the cognate inflammasome sensor inhibited the cell death and IL-18 release. Overall, these data show that IRF1 is dispensable for cell death in response to these inflammasome-activating ligands.

### IRF1 regulates AIM2-dependent PANoptosis

**Figure 3:**
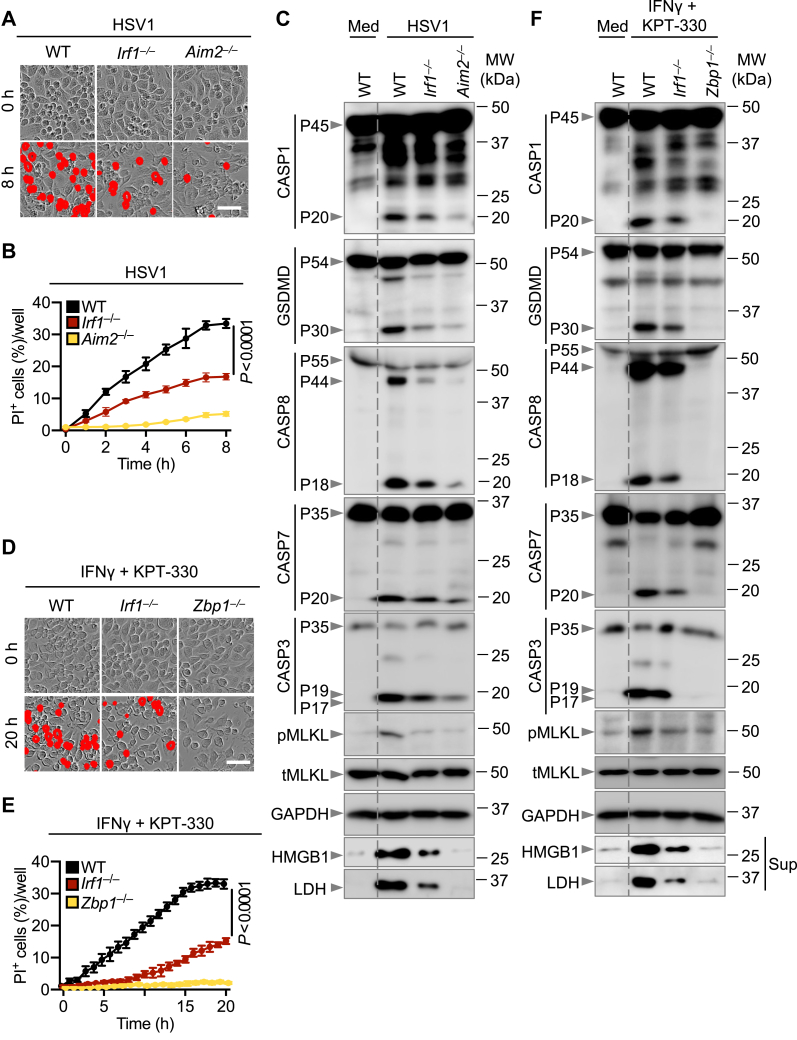
**IRF1 promotes AIM2- and ZBP1-dependent PANoptosis.***A*, representative images of cell death in WT, *Irf1*^*−/−*^, and *Aim2*^*−/−*^ bone marrow–derived macrophages (BMDMs) at 8 h post-infection with HSV1 (10 MOI). *B*, real-time analysis of cell death in WT, *Irf1*^*−/−*^, and *Aim2*^*−/−*^ BMDMs post-infection with HSV1 (10 MOI). *C*, immunoblot analysis of pro- (P45) and activated (P20) caspase-1 (CASP1); pro- (P54) and activated (P30) gasdermin D (GSDMD); pro- (P55) and cleaved (P44 and P18) caspase-8 (CASP8); pro- (P35) and cleaved (P20) caspase-7 (CASP7); pro- (P35) and cleaved (P19 and P17) caspase-3 (CASP3); phosphorylated mixed lineage kinase domain-like (pMLKL) and total MLKL (tMLKL) in HSV1–infected BMDMs at 8 h post-infection or BMDMs in media (Med). Immunoblot analysis of HMGB1 and LDH from the supernatant (Sup) of HSV1-infected BMDMs at 8 h post-infection or BMDMs in Med. *D*, representative images of cell death in WT, *Irf1*^*−/−*^, and *Zbp1*^*−/−*^ BMDMs following stimulation with IFNγ plus KPT-330 at 20 h poststimulation. *E*, real-time analysis of cell death in WT, *Irf1*^*−/−*^, and *Zbp1*^*−/−*^ BMDMs following stimulation with IFNγ plus KPT-330. *F*, immunoblot analysis of pro- and activated CASP1, pro- and activated GSDMD, pro- and cleaved CASP8, pro- and cleaved CASP7, pro- and cleaved CASP3, and pMLKL and tMLKL in IFNγ plus KPT-330–stimulated cells at 20 h poststimulation or BMDMs in Med. Immunoblot analysis of HMGB1 and LDH from the Sup of IFNγ plus KPT-330–stimulated cells at 20 h poststimulation or BMDMs in Med. Data are representative of at least three independent biological replicates. The *dotted lines* in immunoblot panels (*C* and *F*) are incorporated to visually separate media (Med) and treated conditions. Each antibody was probed on an individual blot, and samples from the same experiment were loaded to multiple gels (*C* and *F*). GAPDH was used as a single loading control for each set of samples. Analysis was performed using the two-way ANOVA (*B* and *E*). Scale bar is representative of 50 μm. AIM2, absent in melanoma-2; HMGB1, high mobility group box 1; HSV1, herpes simplex virus type 1; IFN, interferon; IRF1, IFN regulatory factor 1; LDH, lactate dehydrogenase; MOI, multiplicity of infection; ZBP1, Z-DNA binding protein 1.

IRF1 was previously shown to be required for AIM2 inflammasome activation and cell death in response to *F. novicida* infection, whereas it is dispensable in response to transfection with poly(dA:dT), an AIM2-activating ligand (23). Recent studies have shown that the AIM2 inflammasome acts as an integral component of the AIM2-PANoptosome in response to *F. novicida* and HSV1 infections to induce PANoptosis (16). We therefore investigated the role of IRF1 in regulating cell death in response to *F. novicida* and HSV1 infections. Consistent with previous findings, we found that BMDMs deficient in IRF1 showed reduced cell death in response to infection with *F. novicida* (Fig. S1, *A* and *B*). Similarly, we observed reduced cell death in *Irf1*^−/−^ BMDMs compared with WT BMDMs in response to HSV1 infection (Fig. 3, *A* and *B*). Furthermore, loss of IRF1 reduced the activation of PANoptotic molecules, including caspase-1 and gasdermin D (GSDMD), caspase-8, -7, and -3, and MLKL, and the release of DAMPs, such as LDH (lactate dehydrogenase) and HMGB1 (high mobility group box 1), in response to *F. novicida* (Fig. S1*C*) and HSV1 infections (Fig. 3*C*). Because IRF1 is a transcription factor, we assessed the expression of AIM2 *via* both quantitative PCR (qPCR) and Western blotting to determine whether IRF1 mediated its regulatory effects on PANoptosis by regulating the expression of AIM2. We found that IRF1-deficient cells had reduced expression of *Aim2* mRNA and AIM2 protein in response to HSV1 (Fig. S1, *D* and *E*), suggesting that IRF1-mediated transcription of AIM2 is critical for the induction of the AIM2-PANoptosome and PANoptosis. Overall, these data suggest that IRF1 regulates AIM2-mediated PANoptosis by regulating AIM2 expression.

### IRF1 promotes ZBP1-dependent PANoptosis

The combination of IFN and a nuclear export inhibitor, such as KPT-330, induces ZBP1-mediated PANoptosis and regresses tumors in mice (12). Given the role of IRF1 in regulating ZBP1 expression (19) and in mediating PANoptosis in response to sterile innate immune triggers (29), we tested whether IRF1 regulated cell death in response to IFNγ plus KPT-330. We found that loss of IRF1 significantly reduced cell death in response to IFNγ plus KPT-330 (Fig. 3, *D* and *E*). Furthermore, the activation of key PANoptotic molecules, including caspase-1 and GSDMD, caspase-8, -7, and -3, and MLKL, and the release of DAMPs, such as LDH and HMGB1, were reduced in *Irf1*^−/−^ BMDMs compared with WT in response to IFNγ plus KPT-330 (Fig. 3*F*). To further understand the role of IRF1 in regulating ZBP1-dependent PANoptosis, we infected BMDMs with IAV, which triggers the assembly of the ZBP1-PANoptosome to induce cell death (5). We found reduced cell death and reduced activation of PANoptotic molecules as well the release of DAMPs in *Irf1*^−/−^ BMDMs compared with WT BMDMs in response to IAV infection (Fig. S2, *A*–*C*). Similar to our observations that IRF1 regulated AIM2 expression to control AIM2-mediated PANoptosis, we also found that IRF1-deficient cells had reduced expression of *Zbp1* mRNA and ZBP1 protein in response to IAV (Fig. S2, *D* and *E*). Together, these data suggest that IRF1 contributes to ZBP1-mediated PANoptosis in response to stimulation with IFNγ plus KPT-330 and infection with IAV by regulating ZBP1 expression.

### IRF1 induces RIPK1-dependent PANoptosis

**Figure 4:**
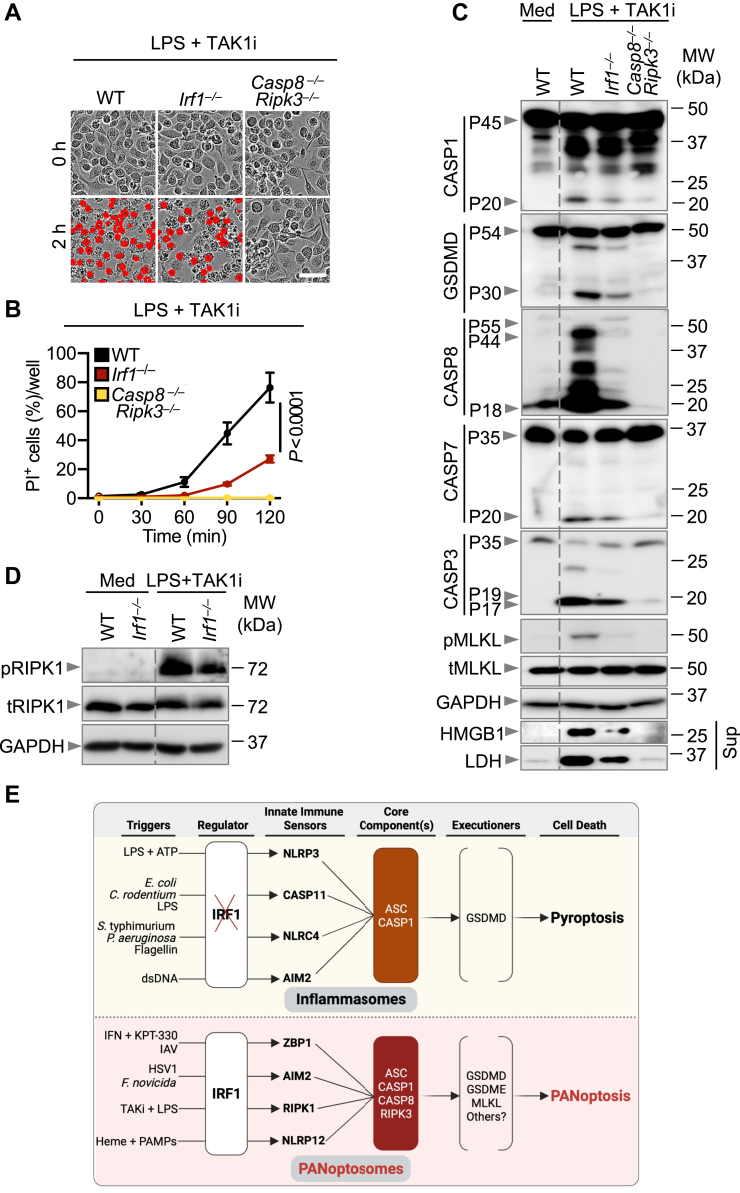
**IRF1 induces RIPK1-dependent PANoptosis.***A*, representative images of cell death in WT, *Irf1*^*−/−*^, and *Casp8*^*−/−*^*Ripk3*^*−/−*^ bone marrow–derived macrophages (BMDMs) at 2 h poststimulation with LPS. BMDMs were primed with TAK1i for 2 h before stimulation with LPS. *B*, real-time analysis of cell death in WT, *Irf1*^*−/−*^, and *Casp8*^*−/−*^*Ripk3*^*−/−*^ BMDMs following TAK1i priming and LPS stimulation. *C*, immunoblot analysis of pro- (P45) and activated (P20) caspase-1 (CASP1); pro- (P54) and activated (P30) gasdermin D (GSDMD); pro- (P55) and cleaved (P44 and P18) caspase-8 (CASP8); pro- (P35) and cleaved (P20) caspase-7 (CASP7); pro- (P35) and cleaved (P19 and P17) caspase-3 (CASP3); phosphorylated mixed lineage kinase domain-like (pMLKL) and total MLKL (tMLKL) in TAK1i-primed and LPS-stimulated cells at 2 h poststimulation with LPS or BMDMs in media (Med). Immunoblot analysis of HMGB1 and LDH from the supernatant (Sup) of TAK1i-primed and LPS-stimulated cells at 2 h poststimulation with LPS or BMDMs in Med. *D*, immunoblot analysis of pRIPK1 and tRIPK1 in TAK1i-primed and LPS-stimulated cells at 2 h poststimulation with LPS or BMDMs in Med. *E*, schematic summary of the proposed function of IRF1 in response to different innate immune triggers that induce inflammasome activation and pyroptosis *versus* PANoptosis. Data are representative of at least three independent biological replicates. The *dotted lines* in immunoblot panels (*C* and *D*) are incorporated to visually separate media (Med) and treated conditions. Each antibody was probed on an individual blot, and samples from the same experiment were loaded to multiple gels (*C* and *D*). GAPDH was used as a single loading control for each set of samples. Analysis was performed using the two-way ANOVA (*B*). Scale bar is representative of 50 μm. HMGB1, high mobility group box 1; IRF1, IFN regulatory factor 1; LDH, lactate dehydrogenase; LPS, lipopolysaccharide; RIPK, receptor interacting protein kinase; TAK1i, transforming growth factor-β activating protein kinase 1 inhibitor.

TAK1 regulates cellular homeostasis and proinflammatory signaling by activating NF-κB and mitogen-activated protein kinase signaling (32). In addition, TAK1 is a central regulator of cell death and inflammation (33, 34), preventing spontaneous NLRP3 inflammasome activation and inflammatory cell death (10). It is now known that TAK1 is a negative regulator of PANoptosis, and that inhibition of TAK1 combined with innate immune priming, such as LPS treatment or bacterial infection, mediates RIPK1-dependent PANoptosis (17, 35). We found that IRF1 was a key regulator of AIM2- and ZBP1-mediated PANoptosis, so we next examined whether IRF1 is a regulator of cell death in response to treatment with a TAK1 inhibitor (TAK1i) plus LPS. We found that IRF1-deficient cells showed reduced cell death compared with WT cells (Fig. 4, *A* and *B*). We then examined whether IRF1 regulated the activation of PANoptotic molecules and found that loss of IRF1 led to a reduction in the activation of caspase-1 and GSDMD, caspase-8, -7 and -3, and MLKL as well as the release of DAMPs (Fig. 4*C*).

Moreover, to understand how IRF1 regulates RIPK1-mediated PANoptosis, we assessed the activation of RIPK1 *via* its phosphorylation. We found that IRF1-deficient cells had reduced activation of RIPK1 when compared with WT cells (Fig. 4*D*). Overall, these data suggest a role for IRF1 in RIPK1-mediated PANoptosis in response to stimulation with TAK1i plus LPS.

Combined with the recent finding that IRF1 also regulates NLRP12-mediated PANoptosis by modulating *Nlrp12* expression (18), these results suggest that IRF1 is a common upstream regulator of ZBP1-, AIM2-, RIPK1-, and NLRP12-PANoptosome formation and PANoptosis (Fig. 4*E*).

## Discussion

IRFs have distinct and critical innate immune functions (36). IRF1 and IRF8 regulate the transcription of several genes required for host defense (37). In addition, IRF9 promotes cell death during IAV infection (5), and IRF8 is required for both ligand- and pathogen-based inflammasome activation and cell death in response to NLRC4-activating stimuli (38). Previous work has shown that only IRF1, but not other IRFs, regulates PANoptosis in response to TNF + IFNγ (29). Given the specific function of IRF1 in PANoptosis in response to this trigger, and the recent discovery that IRF1 regulates the NLRP12-PANoptosome (18), we focused our study on determining whether IRF1 regulates PANoptosis more generally by assessing other conditions that activate the three other known PANoptosome complexes (ZBP1-, AIM2-, and RIPK1-PANoptosomes). We found that IRF1 was dispensable for cell death and IL-18 cytokine release in response to bacterial and ligand-based NLRC4 inflammasome triggers. We also found that IRF1 was dispensable for noncanonical NLRP3 inflammasome triggers, as well as NLRP3 and AIM2 ligand-based triggers, all of which induce pyroptosis. Conversely, IRF1 regulated PANoptosis in response to triggers where the NLRP3, AIM2, or NLRP12 inflammasomes come together with caspase-8 and RIPK3 to act as integral components of the PANoptosome. This suggests that the regulatory role of IRF1 may be a key distinguishing feature between inflammasome formation for pyroptosis and the assembly of a larger PANoptosome complex to drive PANoptosis (Fig. 4*E*).

IRF1 likely regulates PANoptosis through its effects on the transcription of key cell death molecules. For instance, IRF1 regulates the expression of guanylate-binding proteins (GBPs) to induce intracellular bacterial killing and DNA release. GBP2 and GBP5 are required for AIM2 inflammasome formation in response to *F. novicida* infection (39), suggesting they may also be important for the formation of the AIM2-PANoptosome, where the AIM2 inflammasome is an integral component. At the sensor level, IRF1 regulates the expression of ZBP1 in response to IAV (19), and IFN treatment during SARS-CoV-2 and mouse hepatitis virus infections upregulates ZBP1 to drive PANoptosis and cytokine release (40). NLRP12 expression is also regulated by IRF1 in response to heme plus PAMPs or TNF (18). AIM2 is also an IFN-inducible protein (41), and IRF1 regulates its expression during *F. novicida* infection. Furthermore, IRF1 is known to regulate the transcription and protein expression of the cell death executioners GSDMD and MLKL (27, 42, 43), and we observed that it was critical for the expression and activation of key molecules in the PANoptosome. Previous work has shown that deletion of individual cell death effectors does not affect the timing or amplitude of the cell death because of the increased activation of other PANoptotic effectors; it is only by deleting the upstream sensors that are critical for PANoptosome formation that the cell death can be inhibited (11, 16, 17). Therefore, the critical function of IRF1 in regulating these upstream molecules highlights its central role in PANoptosis. However, in many cases, we observed that loss of IRF1 led to a significant, yet incomplete protection from cell death. This partial reduction could be due to functional redundancies between IRF1 and other IRFs or other transcription factors that can upregulate the expression of innate immune molecules, such as NF-κB-mediated transcription.

Overall, our study provides a comprehensive characterization of the role of IRF1 in inflammasome and PANoptosome activation and inflammatory cell death. IRF1 drives ZBP1-, AIM2-, RIPK1-, and NLRP12-dependent PANoptosis in response to infectious and sterile triggers. Furthermore, our data suggest that IRF1 acts as an upstream regulator under conditions where inflammasomes come together with caspase-8 and RIPK3 to form integral components of the PANoptosome but is dispensable for inflammasome formation for pyroptosis. These results suggest that therapies modulating the IRF1 pathway could provide protection against infectious and inflammatory diseases where PANoptosis contributes to pathology.

## Experimental procedures

### Mice

C57BL/6J (WT), *Irf1*^−/−^ (44), *Nlrc4*^*−/−*^ (45), *Casp11*^*−/−*^ (46), *Nlrp3*^*−/−*^ (47), *Aim2*^*−/−*^ (48), *Zbp1*^*−/−*^ (49), and *Casp8*^−/−^*Ripk3*^−/−^ (50) mice have been previously described. WT mice used for this study were bred for three or more generations at the Animal Resources Center at St Jude Children’s Research Hospital, and all knockout mice were backcrossed to these WT mice. The original source of WT mice is The Jackson Laboratory (stock number: 000664). All work with animals was reviewed and approved by the St Jude Children’s Research Hospital Institutional Animal Care & Use Committee.

### Cell culture and stimulation

Primary mouse BMDMs were generated from the bone marrow of WT and the indicated mutant mice. Cells were grown for 5 to 6 days in IMDM (Gibco) supplemented with 1% nonessential amino acids (Gibco), 10% fetal bovine serum (Atlanta Biologicals), 30% L929 conditioned media, and 1% penicillin and streptomycin (Sigma). BMDMs were then seeded in antibiotic-free media at a concentration of 1 × 10^6^ cells into 12-well plates and incubated overnight. The following were used for treatment with innate immune ligand triggers: 50 ng/ml IFNγ (Peprotech; catalog no.: 315-05), 0.1 μm TAK1i (5Z-7-oxozeaeol; Cayman Chemical; catalog no.: 315-05), 100 ng/ml LPS (InvivoGen; catalog no.: 0111: B4 or InvivoGen, tlrl-smlps), and 5 μM KPT-330 (Selleckchem; catalog no.: S7252). For activation of the canonical NLRP3 inflammasome, BMDMs were primed for 4 h with 100 ng/ml ultrapure LPS from *Salmonella minnesota* R595 (InvivoGen; tlrl-smlps) and were stimulated with 5 mM ATP (Roche; catalog no.: 101275310001). For DNA transfection, each reaction consisted of 2 μg poly(dA:dT) (InvivoGen; tlrl-patn) resuspended in PBS and mixed with 0.6 μl Xfect polymer in Xfect reaction buffer (Clontech Laboratories, Inc; catalog no.: 631318). After 10 min, DNA complexes were added to BMDMs in Opti-MEM (Thermo Fisher Scientific; catalog no.: 31985-070). For LPS transfection, the BMDMs were primed for 4 h with 100 ng/ml ultrapure LPS from *E. coli* (Invivogen; catalog no.: 0111: B4) and then transfected with 2 μg of LPS per well following the same steps used for DNA transfection. For flagellin transfection, 0.5 μg of ultrapure flagellin from *Salmonella* Typhimurium (InvivoGen; tlrl-epstfla-5) was resuspended in PBS and mixed with 20 μl of DOTAP (Roche; catalog no.: 11202375001) per reaction. The reaction mixture was incubated for 10 min and added to BMDMs in 500 μl Opti-MEM.

### IL-18 measurement

IL-18 released in the cultured supernatant was measured using ELISA for IL-18 (Invitrogen; catalog no.: BMS618-3) according to the manufacturer’s instructions.

### Virus and bacteria culture

The IAV (A/Puerto Rico/8/34, H1N1 [PR8]) was prepared as previously described (11) and propagated from 11-day-old embryonated chicken eggs by allantoic inoculation. IAV titer was measured by plaque assay in Madin–Darby canine kidney cells. Human herpes simplex virus 1 (HF strain) (American Type Culture Collection [ATCC]; catalog no.: VR-260) was propagated in Vero cells, and the virus titer was measured by plaque assay in Vero cells. *F. novicida* strain U112 was grown in BBL Trypticase soy broth (BD; catalog no.: 211768) supplemented with 0.2% l-cysteine (Thermo Fisher Scientific; catalog no.: BP376-100) overnight under aerobic conditions at 37 °C. Bacteria were subcultured (1:10) in fresh Trypticase soy broth supplemented with 0.2% l-cysteine for 4 h and resuspended in PBS. *Salmonella enterica serovar* Typhimurium (*S. typhimurium*) strain SL1344, *C. rodentium* (ATCC; catalog no.: 51459), and *E. coli* (ATCC; catalog no.: 11775) were inoculated into Luria–Bertani media (MP Biomedicals; catalog no.: 3002-031) and incubated overnight under aerobic conditions at 37 °C. *S*. Typhimurium SL1344 was subcultured (1:10) into fresh LB media for 3 h at 37 °C to generate log phase grown bacteria. For bacterial infection, *F. novicida* (multiplicity of infection [MOI] 100), *S.* Typhimurium (MOI 1), *C. rodentium* (MOI 20), and *E. coli* (MOI 20) were used. Four hours after infection, *E. coli*, *C. rodentium*, and *F. novicida* infected cells were washed two times with PBS, and 50 μg/ml gentamicin (Thermo Fisher Scientific; catalog no.: 15750-060) was added to kill extracellular bacteria. For virus infection, IAV (MOI 20) and HSV (MOI 10) were used in serum-free media; 1 h after infection, 10% fetal bovine serum was added to the cells.

### Real-time imaging for cell death

The kinetics of cell death were determined using the IncuCyte S3 (Sartorius) live-cell automated system. BMDMs (5 × 10^5^ cells/well) in 24-well tissue culture plates and (1 × 10^6^ cells/well) in 12-well tissue culture plates were treated or infected with the indicated innate immune triggers and stained with propidium iodide (Life Technologies; catalog no.: P3566) following the manufacturer’s protocol. The plate was scanned, and fluorescent and phase-contrast images (four image fields/well) were acquired in real-time. Propidium iodide–positive dead cells are marked with a red mask for visualization. The image analysis, masking, and quantification of dead cells were done using the software package supplied with the IncuCyte imager.

### Immunoblot analysis

Cell lysates were combined in caspase lysis buffer (containing protease inhibitors, phosphatase inhibitors, 10% NP-40, and 25 mM DTT) and sample loading buffer (containing SDS and 2-mercaptoethanol) for immunoblot analysis of caspases. For immunoblot analysis of signaling components, supernatants were removed, and cells were washed once with PBS, followed by lysis in radioimmunoprecipitation assay buffer and sample loading buffer. For immunoblot analysis of LDHA and HMGB1, cell supernatant was combined with sample loading buffer (containing SDS and 2-mecrcaptoethanol) at a ratio of 1:1. Proteins were separated by electrophoresis through 8 to 12% polyacrylamide gels. Following electrophoretic transfer of proteins onto polyvinylidene difluoride membranes (Millipore; catalog no.: IPVH00010), nonspecific binding was blocked by incubation with 5% skim milk, then membranes were incubated with primary antibodies against: caspase-3 (Cell Signaling Technology [CST]; catalog no.: 9662), cleaved caspase-3 (CST; catalog no.: 9661), caspase-7 (CST; catalog no.: 9492), cleaved caspase-7 (CST; catalog no.: 9491), caspase-8 (CST; catalog no.: 4927), cleaved caspase-8 (CST; catalog no.: 8592), caspase-1 (AdipoGen; catalog no.: AG-20B-0042), GAPDH (CST; catalog no.: 5174), phosphorylated MLKL (CST; catalog no.: 37333), total MLKL (Abgent; catalog no.: AP14242B), GSDMD (Abcam; catalog no.: 209845), AIM2 (CST; catalog no.: 53491), ZBP1 (AdipoGen; catalog no.: AG-20B-0010), pRIPK1 (CST; catalog no.: 311222), tRIPK1 (CST; catalog no.: 3493S), HMGB1 (Abcam; catalog no.: 18256), and LDHA (Proteintech; catalog no.: 19987-1-AP). Membranes were then washed and incubated with the appropriate horseradish peroxidase–conjugated secondary antibodies (Jackson ImmunoResearch Laboratories; anti-rabbit [catalog no.: 111-035-047] and anti-mouse [catalog no.: 315-035-047]). Proteins were visualized using Immobilon Forte Western Horseradish Peroxidase Substrate (Millipore; catalog no.: WBLUF0500).

### Quantitative real-time PCR analysis

Quantitative real-time PCR (qRT–PCR) analysis was performed as described previously (51). In brief, RNA was extracted from *in vitro* or *in vivo* samples by using TRIzol (Thermo Fisher Scientific; catalog no.: 155960260) or a Mini-Prep Kit (Bio Basic; catalog no.: BS822322), respectively, in accordance with the manufacturer’s instructions. The isolated RNA was reverse-transcribed into complementary DNA with a First-Strand complementary DNA Synthesis Kit (Applied Biosystems; catalog no.: 4368814). qRT-PCR was performed on an ABI 7500 RT–PCR instrument, using 2× SYBR Green (catalog no.: 4368706; Applied Biosystems) and the appropriate primers. The sequences for the qRT–PCR primers are listed in Table S1.

### Statistical analysis

GraphPad Prism, version 8.0 (GraphPad Software, Inc) was used for data analysis. Data were plotted and provided as mean ± SD. The statistical significance was calculated using two-way ANOVA (with Dunnet’s and Sidak’s multiple comparisons tests). *p* < 0.05 was considered statistically significant. Exact *p* values for all statistically significant comparisons are shown in the graphs. Information about the number of experimental repeats is provided in the corresponding figure legends.

## Data availability

All data used in this article are included within the figures and Supporting information files.

## Supporting information

This article contains supporting information.

## Conflict of interest

T.-D. K. was a consultant for Pfizer. All other authors declare that they have no conflicts of interest with the contents of this article.
