# Supplementary material for: Immune regulator IRF1 contributes to ZBP1-, AIM2-, RIPK1-, and NLRP12-PANoptosome activation and inflammatory cell death (PANoptosis)
Source: J Biol Chem. 2023 Aug 7;299(9):105141. doi: 10.1016/j.jbc.2023.105141 (PMC10494469; doi:10.1016/j.jbc.2023.105141)
Supplement: Supporting Figures S1 and S2 and Table S1 [file mmc1.pdf]

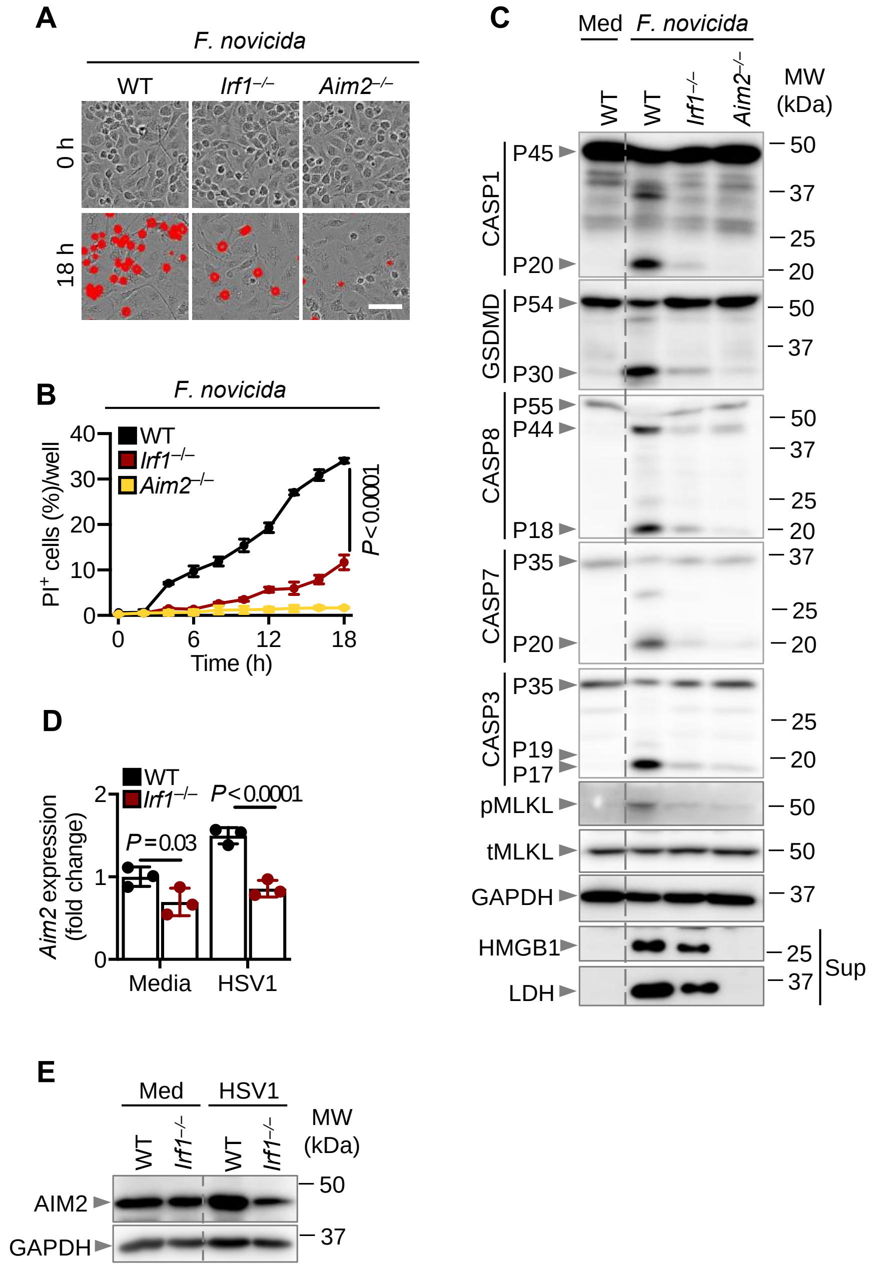

Figure S1. IRF1 promotes AIM2-dependent PANoptosis in response to infection with *Francisella novicida*

(**A**) Representative images of cell death in WT**,** *Irf1*$^{–/–}$**,** and *Aim2*$^{–/–}$ bone marrow-derived macrophages (BMDMs) at 18 h post-infection with *F. novicida* (100 MOI). (**B**) Real-time analysis of cell death in WT**,** *Irf1*$^{–/–}$**,** and *Aim2*$^{–/–}$ BMDMs following infection with *F. novicida* (100 MOI). (**C**) Immunoblot analysis of pro- (P45) and activated (P20) caspase-1 (CASP1); pro- (P54) and activated (P30) gasdermin D (GSDMD); pro- (P55) and cleaved (P44 and P18) caspase-8 (CASP8); pro- (P35) and cleaved (P20) caspase-7 (CASP7); pro- (P35) and cleaved (P19 and P17) caspase-3 (CASP3); phosphorylated mixed lineage kinase domain-like (pMLKL) and total MLKL (tMLKL) in *F. novicida*-infected BMDMs at 18 h post-infection or BMDMs in media (Med). Immunoblot analysis of HMGB1 and LDH from the supernatant (Sup) of *F. novicida*-infected BMDMs at 18 h post-infection or BMDMs in Med. (**D**) Transcript level of *Aim2* in WT and *Irf1*$^{–/–}$ BMDMs infected with HSV1 for 4 h. (**E**) Immunoblot analysis of AIM2 in WT and *Irf1*$^{–/–}$ BMDMs infected with HSV1 for 8 h or BMDMs in Med. Data are representative of at least three independent biological replicates. The dotted lines in immunoblot panels **(C, E)** are incorporated to visually separate media (Med) and treated conditions. Each antibody was probed on an individual blot, and samples from the same experiment were loaded to multiple gels **(C, E)**. GAPDH was used as a single loading control for each set of samples. Analysis was performed using the two-way ANOVA (**B, D**). Scale bar is representative of 50 μm.

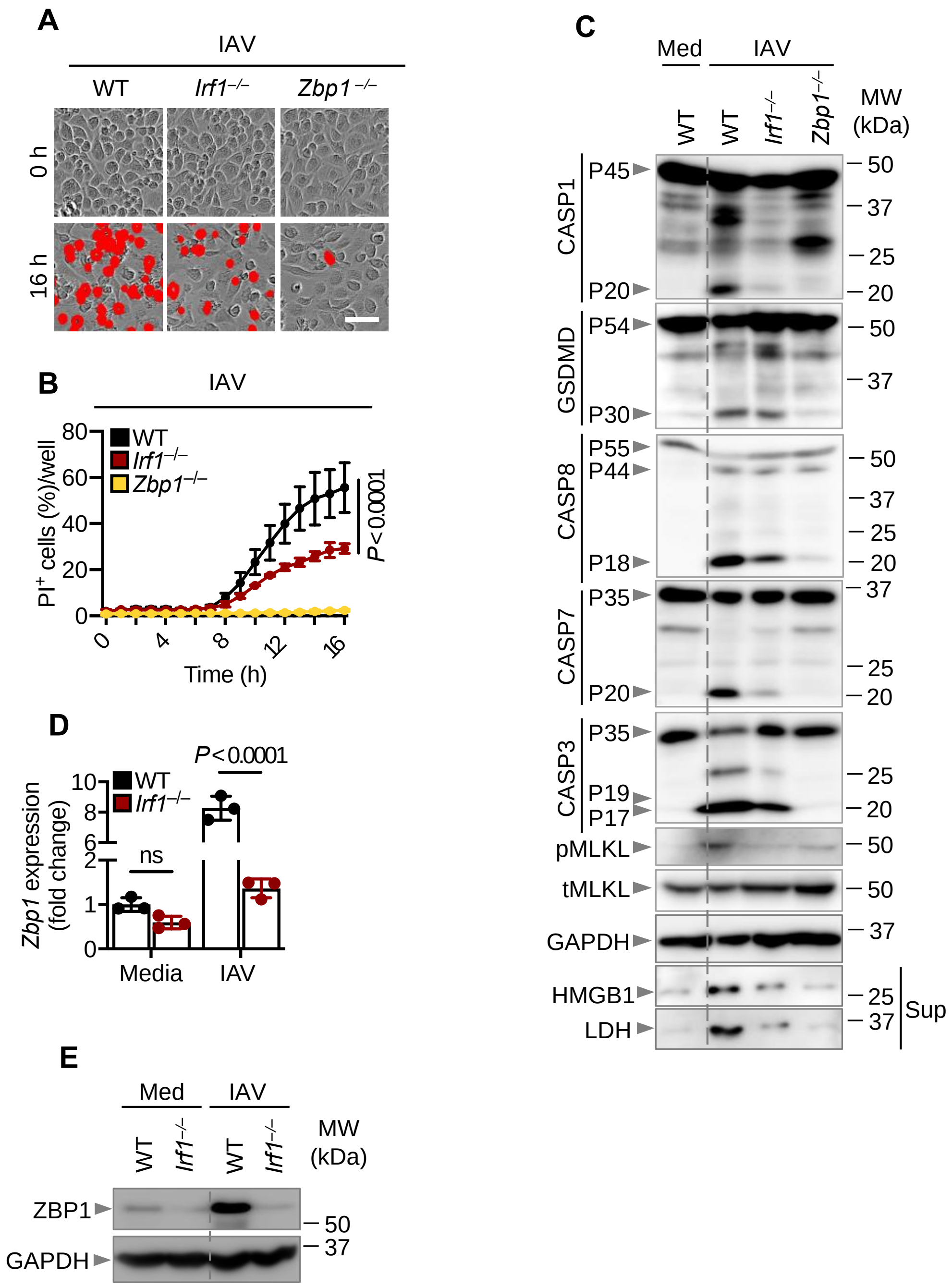

Figure S2. IRF1 promotes ZBP1-dependent PANoptosis in response to infection with IAV
(**A**) Representative images of cell death in WT**,** *Irf1*$^{-/-}$**,** and *Zbp1*$^{-/-}$ bone marrow-derived macrophages (BMDMs) at 16 h post-infection with IAV (20 MOI). (**B**) Real-time analysis of cell death in WT**,** *Irf1*$^{-/-}$**,** and *Zbp1*$^{-/-}$ BMDMs following infection with IAV (20 MOI). (**C**) Immunoblot analysis of pro- (P45) and activated (P20) caspase-1 (CASP1); pro- (P54) and activated (P30) gasdermin D (GSDMD); pro- (P55) and cleaved (P44 and P18) caspase-8 (CASP8); pro- (P35) and cleaved (P20) caspase-7 (CASP7); pro- (P35) and cleaved (P19 and P17) caspase-3 (CASP3); phosphorylated mixed lineage kinase domain-like (pMLKL) and total MLKL (tMLKL) in IAV-infected BMDMs at 16 h post-infection or BMDMs in media (Med). Immunoblot analysis of HMGB1 and LDH from the supernatant (Sup) of IAV-infected BMDMs at 16 h post-infection. (**D**) Transcript level of *Zbp1* in WT and *Irf1*$^{-/-}$ BMDMs infected with IAV for 9 h. (**E**) Immunoblot analysis of ZBP1 in WT and *Irf1*$^{-/-}$ BMDMs infected with IAV for 16 h or BMDMs in Med. Data are representative of at least three independent biological replicates. The dotted lines in immunoblot panels **(C, E)** are incorporated to visually separate media and treated conditions. Each antibody was probed on an individual blot, and samples from the same experiment were loaded to multiple gels **(C, E)**. GAPDH was used as a single loading control for each set of samples. Analysis was performed using the two-way ANOVA (**B, D**). Scale bar is representative of 50 µm.

Supplementary Table 1. Real-time qPCR primer sequences

Target	Primer sequence
mZbp1	Forward: 5’-GACGACAGCCAAAGAAGTGA-3’<br>Reverse: 5’-GAGCTATGTCTTGGCCTTCC-3’
mAim2	Forward: 5’-GAT TCA AAG TGC AGG TGC GG -3’<br>Reverse: 5’-TCT GAG GCT TAG CTT GAG GAC-3’
mActin	Forward: 5’-GTGTGACGTTGACATCCGTA -3’<br>Reverse: 5’-CACAGAGTACTTGCGCTCAG -3’
